# Association of the blood eosinophil count with end-organ symptoms

**DOI:** 10.1016/j.amsu.2019.06.015

**Published:** 2019-07-09

**Authors:** Ole Weis Bjerrum, Volkert Siersma, Hans Carl Hasselbalch, Bent Lind, Christen Lykkegaard Andersen

**Affiliations:** a Department of Hematology, Copenhagen University Hospital, Rigshospitalet, Denmark; b Department of Hematology, Odense University Hospital, Denmark; c The Research Unit for General Practice and Section of General Practice, Department of Public Health, University of Copenhagen, Denmark; d Department of Hematology, Roskilde University Hospital, Denmark; e Department of Clinical Biochemistry, Hvidovre University Hospital, Denmark

**Keywords:** Eosinophilia, Epidemiology, Haematology

## Abstract

**Introduction:**

Eosinophilia may cause organ dysfunction, but an exact relation between eosinophil blood counts and adverse outcomes has not been described. The aim of the study is to associate in one model both normal and increased blood eosinophil counts to the subsequent development of common conditions in internal medicine, in which eosinophil granulocytes may play a role for the symptoms.

**Methods:**

From the Copenhagen Primary Care Differential Count (*CopDiff*) Database, we identified 359,950 individuals with at least one differential cell count (DIFF) during 2000–2007. From these, one DIFF was randomly chosen. From the Danish National Patient Register we ascertained organ damage, within four years following the DIFF. Using multivariable logistic regression, odds ratios were calculated and adjusted for previous eosinophilia, sex, age, year, month, CRP and comorbid conditions.

**Results:**

Risks for skin- and respiratory disease were increased from above the median eosinophil count of 0.16 × 10^9^/l and reached a plateau around 1.0 × 10^9^/l. Furthermore, risks of most outcomes also increased when the eosinophil count approached zero.

**Conclusions:**

The observed U-shaped association with a plateau of risks around 1 × 10^9^/l indicates that the risk for symptoms due to eosinophilia do not increase proportionate at higher counts. This study demonstrates for the first time that there is indeed an increased risk below median count of 0.16 × 10^9^/l for an increased risk for the same manifestations. Clinically, it means that a normal or even low count of eosinophils do not rule out a risk for organ affection by eosinophils, and may contribute to explain, why patients may have normal eosinophil counts in e.g. asthma or allergy and still have symptoms from the lungs and skin, most likely explained by the extravasation of eosinophils.

## Highlights

• Blood eosinophilia may cause end-organ symptoms.
• An exact relation between eosinophil count and outcome has not been demonstrated.
• Eosinophil numbers correlate to organ damage even below the definition of eosinophilia.
• This association is U-shaped between organ manifestations and eosinophil count in blood.
• A plateau of risks is observed around 1 × 10^9^/l.

## 1 Introduction

In healthy individuals, eosinophilic granulocytes (eosinophils) constitute less than five percent of all white blood cells [1]. Blood eosinophilia, traditionally defined for use in clinical practice as an eosinophil count of ≥0.5 × 10^9^/l, is encountered in all areas of medicine and in both primary and secondary care. It may arise from either clonal intrinsic disorders or from reactive extrinsic conditions [[2], [3], [4]]. Reactive causes account for the vast majority of cases. A plethora of distinct disease entities with concomitant eosinophilia has been known for many years, while the primary eosinophilic conditions were not introduced until 1968 [1,[5], [6], [7]].

For the prognostic evaluation and management of patients presenting with eosinophilia it is important to identify both the many patients with reactive eosinophilia and those patients with the rarer specific clonal diseases. This leaves a very small subgroup of patients with *idiopathic hypereosinophilia* [3,4,7,8], where neither clonality nor other primary stimuli can be demonstrated. Several useful algorithms for such workup have been presented.

The eosinophilic granulocyte may have diverse physiological functions, which in principle are beneficial in the immune reaction against exogenous (infections) and endogenous (inflammation and cancer) intruders [9,10]. However, irrespective of the cause of eosinophilia, the activation of eosinophils may also result in inappropriate organ involvement due to tissue invasion and release of cytokines, peptides, metabolites and proteinases from the granule matrix or cell surface [1,2]. Accordingly, organ involvement from both clonal and reactive causes has been reported and such deleterious effects may be one of the initial manifestations of eosinophil-related disease [3,7,9,10].

So far, no association between levels of blood eosinophils and organ manifestations has been demonstrated. However, it seems as if eosinophils show a predilection for certain organ systems such as heart [[11], [12], [13], [14], [15], [16], [17]], lungs [[18], [19], [20], [21], [22], [23]], gastrointestinal system [[24], [25], [26], [27]], nervous system [[28], [29], [30], [31], [32], [33], [34], [35]] and skin [[36], [37], [38], [39], [40]]. A scoring system guiding therapy based on certain paraclinical determinations was introduced some thirty years ago for patients with *idiopathic hypereosinophilic syndrome* [41,42], but this has not been implemented in clinical work, and today the degree of blood eosinophilia as mild (≥0.5 × 10^9^/l-1.5 × 10^9^/l), moderate (≥1.5 × 10^9^/l-5.0 × 10^9^/l) and severe (≥5.0 × 10^9^/l) is arbitrary and not based on risk stratification for organ manifestations [1,3].

This is the first study to examine both normal and increased number of blood eosinophils and the development of various common medical conditions. The rationale for this study was to investigate the number of eosinophil granulocytes in blood samples and the subsequent risk in the same individual subjects in a large population-based cohort to develop disorders with organ involvement, where eosinophils may play a pathophysiological role, in order to reflect a functional context, which has not been established previously.

## 2 Methods

**Fig. 1:**
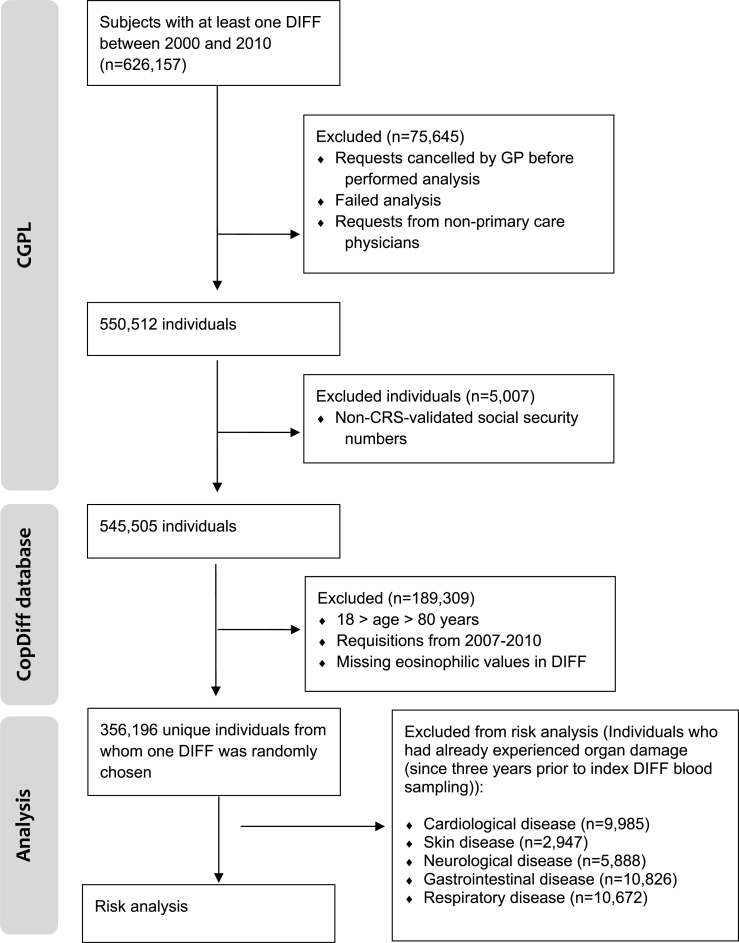
Flowchart. CGPL, Copenhagen General Practitioners' Laboratory; CopDiff, Copenhagen Primary Care Differential Count; CRS, The Danish Civil Registration System; DIFF, differential cell count; GP, general practitioner.

The Copenhagen General Practitioners’ Laboratory (CGPL) served the general practitioners (GPs) in the Copenhagen area up until 2016 and covered approximately 1.3 million inhabitants. CGPL had International Organization for Standardization (ISO) accreditation and has registered all analytical results since May 1, 2000. The Copenhagen Primary Care Differential Count (*CopDiff*) database contains results from all differential cell counts (DIFFs) requested by GPs in Copenhagen from July 1, 2000 to January 25, 2010. From each of the 359,950 unique individuals (aged 18–80 years) with at least one DIFF in the period January 1, 2001 to December 31, 2007, a single DIFF encompassing the eosinophil count was randomly chosen by computer-generated random numbers (n = 356,196; Fig. 1). Eosinophil test results reported as “<0.02 × 10^9^/l” were set to “0.0 × 10^9^/l” in order to maintain only numeric values in the database (n = 1889). Where available, the level of C-reactive-protein (CRP), categorized as “increased” (≥10 mg/l) vs. “normal” (<10 mg/l) was also obtained from the database (n = 229,511). Furthermore, we recorded whether another DIFF was made during the 6 months before our request (n = 32,475) and whether eosinophilia was present in this DIFF. In November 2013, the *CopDiff* database was linked to The Danish National Patient Register (NPR) which has recorded information on all contacts since 1977 with hospitals in Denmark, including discharge diagnoses, outpatient clinic contacts and surgical procedures performed; and to which reporting is mandatory by law in Demark [43].

Outcomes was potentially eosinophil-related organ damage (taken from the NPR) over the 4-year period following the DIFF defined according to the ICD nomenclature and grouped as: *“Cardiac disease”, “Skin disease”, “Neurological disease”, “Gastrointestinal disease”* and *“Respiratory disease”*. Please refer to the Supplementary Table 1 for details on these entities and references. To adjust for possible confounding by comorbid conditions, we also computed Charlson's Comorbidity Index (CCI) [44] from the hospital contacts recorded in the NPR for three years before the DIFF. The study was approved by the Danish Data Protection Agency. According to Danish legislation no ethical approval or patient consent was required since the patients were not approached at any time during the conduct of the study However, it is not possible without access to each individual patient file to determine the time elapsed from symptoms to blood sampling to diagnosis.

## 3 Statistical analysis

We used multivariable logistic regression to calculate the odds ratio (OR) for the 4 -year incidences of the outcomes between the eosinophil count and a baseline count of 0.16 × 10^9^/l which was the median eosinophil count in our data. This OR was adjusted for previous eosinophilia, sex, age, year, month, CRP and competing comorbid conditions (CCI), and modelled as a restricted cubic spline [45]. In order to assess only *de novo* cases of potential eosinophil-related end-organ damage, individuals who had already experienced organ damage (since three years prior to index DIFF blood sampling) were excluded from risk analyses. A Chi-squared test was used for comparison of the observed distributions of incident disease within the five organ damage groups between the eosinophil groups of *“* < *0.16x10*^*9*^*/l”* and *“*≥*0.16x10*^*9*^*/l”*. All analyses and calculations were performed with SAS version 9.4 (SAS Institute Inc., Cary, NC, USA). This study was registered by The Danish Data Protection Agency J.nr. 2013-231-0053 and by www.researchregistry.com UIN 4955.

## 4 Results

In the full cohort of 359,950 individuals there was a female/male sex ratio of 1.38 (208,691/151,259) and a mean age (SD) of 48.3 (16.7) years. Of these, 14,406 individuals (4%) had eosinophilia (≥0.5 × 10^9^/l). Compared with the baseline count of 0.16 × 10^9^/l which was the median eosinophil count in our data, risks for skin- and respiratory disease were increased both above and below the definition of eosinophilia (Table 1). At the 99th percentile, corresponding to an eosinophil count of 0.75 × 10^9^/l, risks of respiratory end-organ damage were increased more than two-fold with OR (95% C.I.) of 2.11 (1.96–2.27, P < 0.001). The corresponding risk increase estimate for skin disease was 1.88 (1.64–2.15, P < 0.001).

**Table 1:** Odds ratios (ORs) for the four-year incidence of eosinophil-related end-organ-damage for selected percentiles.

Percentile	Eosinophils (10^9^/l)	Cardiac disease				Skin disease				Neurological disease				Gastrointestinal disease				Respiratory disease			
			95% C.I.				95% C.I.				95%CI				95% C.I.				95% C.I.		
		Odds ratio	Lower	Upper	P-value	Odds ratio	Lower	Upper	P-value	Odds ratio	Lower	Upper	P-value	Odds ratio	Lower	Upper	P-value	Odds ratio	Lower	Upper	P-value
1%	0.02	1.19	1.10	1.28	<0.001	1.16	1.01	1.34	0.038	1.12	1.02	1.24	0.022	1.19	1.11	1.28	<0.001	1.00	0.92	1.08	0.959
2%	0.03	1.15	1.08	1.23	<0.001	1.12	0.99	1.26	0.061	1.11	1.02	1.20	0.019	1.16	1.09	1.23	<0.001	0.98	0.91	1.05	0.518
5%	0.05	1.10	1.05	1.15	<0.001	1.05	0.97	1.14	0.205	1.07	1.01	1.14	0.014	1.11	1.06	1.15	<0.001	0.94^*^	0.90	0.99	0.022
10%	0.07	1.06	1.03	1.09	<0.001	1.01	0.96	1.07	0.726	1.05	1.01	1.09	0.015	1.06	1.03	1.10	<0.001	0.93^*^	0.90	0.96	<0.001
25%	0.10	1.02	1.00	1.04	0.024	0.98	0.95	1.01	0.165	1.02	1.00	1.05	0.044	1.02	1.01	1.04	0.004	0.93^*^	0.91	0.94	<0.001
50%	0.16	1.00	–	–	–	1.00	–	–	–	1.00	–		–	1.00	–	–	–	1.00	–		–
75%	0.26	1.04	1.01	1.07	0.017	1.15	1.09	1.22	<0.001	1.01	0.97	1.06	0.512	1.05	1.01	1.08	0.005	1.24	1.20	1.28	<0.001
90%	0.38	1.06	1.02	1.11	0.004	1.27	1.18	1.37	<0.001	1.06	1.00	1.12	0.034	1.12	1.07	1.16	<0.001	1.49	1.43	1.55	<0.001
95%	0.47	1.06	1.00	1.12	0.041	1.34	1.21	1.48	<0.001	1.10	1.02	1.18	0.009	1.16	1.10	1.22	<0.001	1.64	1.56	1.73	<0.001
98%	0.62	1.06	0.98	1.14	0.138	1.55	1.37	1.76	<0.001	1.14	1.04	1.25	0.005	1.21	1.13	1.30	<0.001	1.89	1.77	2.02	<0.001
99%	0.75	1.06	0.98	1.15	0.166	1.88	1.64	2.15	<0.001	1.16	1.04	1.28	0.007	1.23	1.14	1.34	<0.001	2.11	1.96	2.27	<0.001

Values are percentiles, eosinophil counts, odds ratios, 95% confidence intervals and P-values for the defined outcomes from multivariate logistic regression analysis and adjusted for previous eosinophilia, sex, age, year, month, CRP and Charlson's Comorbidity Index. * This apparent protective effect is a consequence of the chosen baseline eosinophil count of 0.16 × 10^9^/l and does not reflect a true protective effect.

Odds ratios of 2 may be interpreted given the number of specific patients in Table 2. Notwithstanding this, the model to obtain results included logistic regression and adjustments as described to capture only *de novo* cases.

**Table 2:** The distribution of incident cases of disease (within 4 years from DIFF) in eosinophil groups.

Type	Eosinophils <0.16 × 10^9^/l, n	Percent within group	Eosinophils ≥0.16 × 10^9^/l, n	Percent within group
Cardiac disease, individuals at risk = 346,211^a^				
Pericardium	124	1.7%	172	1.8%
Endocardium	61	0.9%	43	0.5%
Valve	510	7.2%	713	7.6%
Myocardium	146	2.1%	175	1.9%
Conduction	3916	55.2%	4997	53.0%
Heart failure	1812	25.6%	2653	28.2%
Other heart disease	521	7.3%	671	7.1%
Total, P = 0.0002^†^	7090	100.0%	9424	100.0%
Skin disease, individuals at risk = 353,249^a^				
Dermatitis and eczema	1.427	76.3%	1933	77.6%
Urticaria and erythema	444	23.7%	557	22.4%
Total, P = 0.29^†^	1871	100.0%	2490	100.0%
Neurological disease, individuals at risk = 350,308^a^				
Degenerative diseases of the nervous system	545	13.6%	587	10.8%
Mononeuritis multiplex	6	0.1%	17	0.3%
Polyneuropathies	812	20.3%	1065	19.6%
Paralytic syndromes	175	4.4%	247	4.5%
Encephalopathy, unspecified	16	0.4%	27	0.5%
Cerebrovascular disease	2447	61.2%	3501	64.3%
Total, P = 0.0004^†^	4001	100.0%	5444	100.0%
Gastrointestinal disease, individuals at risk = 345,370^a^				
Diseases of esophagus, stomach and duodenum	4100	53.9%	5239	55.5%
Non-infective enteritis and colitis	1668	21.9%	2170	23.0%
Diseases of liver	1235	16.3%	1298	13.7%
Disorders of gallbladder, biliary tract and pancreas	597	7.9%	738	7.8%
Total, P < 0.0001^†^	7600	100.0%	9445	100.0%
Respiratory disease, individuals at risk = 345,524^a^				
Chronic lower respiratory disease	4811	83.2%	8045	85.5%
Respiratory disease principally affecting the interstitium	429	7.4%	619	6.6%
Pleural disease	540	9.3%	743	7.9%
Total, P = 0.0006^†^	5780	100.0%	9407	100.0%

a In order to assess only *de novo* cases of potential eosinophil-related end-organ damage, individuals who had already experienced organ damage (since three years prior to index DIFF blood sampling) were excluded from analyses, please refer to Fig. 1 for details.^†^ Chi-squared test for the overall comparison of distributions between the groups.

**Fig. 2:**
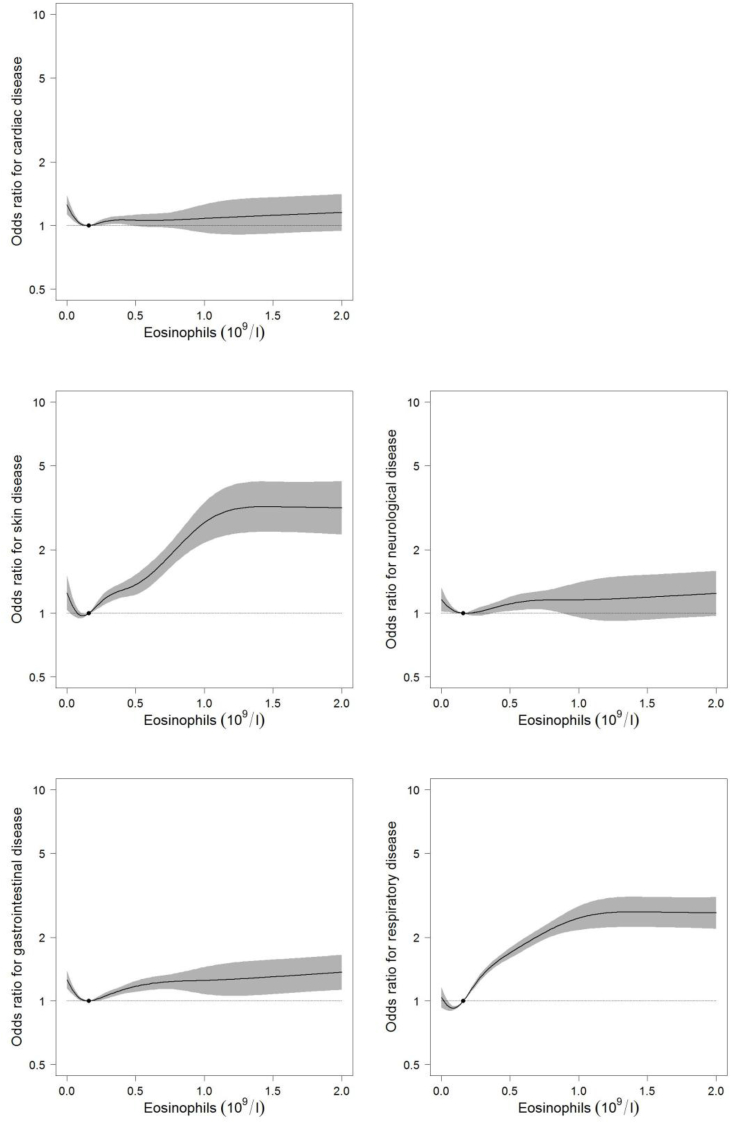
Odds ratio (OR) for the four-year incidence of potential eosinophil-related end-organ-damage for the indicated eosinophil count compared to a baseline count of 0.16 × 10^9^/l (the median eosinophil count in our data). The shaded area around the line denotes the 95% confidence interval.

Furthermore, risks of cardiac, neurological and gastrointestinal disease also increased below the median eosinophil count. To illustrate this non-linear relationship, we used restricted cubic splines of the ORs for the outcomes according to the eosinophil count (Fig. 2). These risk curves were U-shaped for all outcomes and the median eosinophil count of 0.16 × 10^9^/l represented the lowest risk for most outcomes. In addition, all risks reached a plateau at an eosinophil count around 1.0 × 10^9^/l, above which the risks did not increase noticeably.

We then compared incident diagnoses below and above the median eosinophil count of 0.16 × 10^9^/l in an attempt to unmask the mechanisms behind the observed increases in risk for low eosinophil counts. Although overall differences were statistically significant for all groups besides *“Skin disease”,* and this was most likely due to large numbers, no marked differences in frequency distributions were observed (Table 2).

## 5 Discussion

In this study on almost 360,000 individuals, we demonstrate that irrespective of the definition of eosinophilia, eosinophil numbers associate with the subsequent diagnosis of a potential eosinophil-related skin- or respiratory condition even below such a threshold, and that these risks reach a plateau around approximately 1.0 × 10^9^/l. It is possible that these observations explain why a clear relationship between eosinophilia and eosinophil related end-organ damage and prognosis has previously been difficult to demonstrate [41,42]. It is important to bear in mind that a eosinophil blood measurement represents a balance between production and cell turnover, but does not take into account the extravasation of eosinophils from blood to tissues, where the cell perform its functions without returning to the circulation. Hence, the upper normal range of 0.5 eosinophils x 10^9^/l is arbitrary in the context of organ involvement. Therefore, the association of eosinophil counts in individual patients to pathophysiologic relations has to be based on the observed median value, not the normal distribution. In this large population, the median eosinophil count of 0.16 × 10^9^/l was applied as reference to assess cases of potential eosinophil-related end-organ manifestations. Interestingly, the exact same median value was observed in a Dutch population of 13,301 subjects studied for complex metabolic and pulmonary traits and diseases [46].

The plateau of risks of potential eosinophil-related end-organ symptoms around 1.0 × 10^9^/l is important for the management of patients with both reactive and clonal eosinophilia since mild-to-moderate eosinophilia, according to traditional definitions, associates with similar risks of subsequent adverse events as severe eosinophilia. It is important to note that this study is not able to determine the impact of medical intervention on the observed risks. Akin to these observations, a non-linear platelet binding to von-Willebrand factor is observed in thrombocytosis [47], and it is also known that patients with essential thrombocythaemia do not necessarily exhibit higher risks of thrombosis with increasing platelet counts *per se* [48,49].

Increases in risks of skin and respiratory disease were much greater than for the remaining outcomes which were negligible. Such variation could be an expression of the many different risk factors involved in the development of the different outcomes in this study where, as an example, eosinophil-associated cardiac disease constitutes an insignificant proportion of all cardiac outcomes. The implementation of the Charlson's Comorbidity Index to adjust for competing comorbid conditions only allows for rudimentary adjustment for non-eosinophil-related disease. Decreased risks of respiratory disease with low eosinophil counts are observed in our results, however, this finding is a consequence of the chosen baseline eosinophil count of 0.16 × 10^9^/l and does not reflect a protective effect.

All analyses share the U-shaped dose-response relationship (Fig. 2), also termed *hormesis* [50]. These U-shaped curves have been reported for various endpoints of considerable significance to public health, such as longevity and cancer incidence. In all cases it seems as if either too much or too little of a certain stimulus is associated with sub-optimal biological performance [51,52]. In the present setting, the detrimental effect of low eosinophil levels on risks of eosinophil-related end-organ symptoms may reflect the biological process where eosinophils leave the peripheral blood to enter the tissues in order to perform their physiological tasks. This would mimic the extravasation of neutrophils in infection [53,54]. This would also be in accordance with the demonstration of eosinophils in tissue specimens as part of the diagnostic criteria in vasculitis [55] and the inflammatory process of atopic dermatitis [40] and asthma [23]. Hence, a suspected phenomenon of extravasation is important, because it converts the eosinophil count into a prognostic factor for potential end-organ involvement.

We did not observe outspoken differences in diagnoses when comparing the distributions below and above the median eosinophil count (Table 2), but odds ratio for the four-year incidence of potential eosinophil-related end-organ-damage for the indicated eosinophil count compared to the median eosinophil count showed very different relations (Fig. 2).

This study was designed to isolate the possible association, and thus an impact on the predilection for certain organs, of the number of eosinophil granulocytes measured in a blood sample, in a statistical model in a very large well-defined cohort. Other blood cells than eosinophils may also leave the circulation by diapedesis, like neutrophil granulocytes, whereas thrombocytes remain in the circulation. However, these blood cells have different turnover and cellular dynamic and make it difficult to interpret the functional interplay of eosinophils and other cells in the individual subject, analysed in one blood sample at a given time point.

The *CopDiff* database has some important strengths. Firstly, access to all DIFFs from all GPs on some 360,000 individuals from the Copenhagen area over a 10-year period offers a unique insight from a population sample that covers approximately 20% of the entire population of Denmark. Furthermore, the *CopDiff* population was sampled continuously without any restrictions as to why the DIFF was requested by the GP. This, together with the use of a computer-generated random selection among these DIFFs, diminishes selection bias which would have been more likely if opting for, for instance, “the first DIFF” or “the DIFF closest to an outcome of interest”. Secondly, all diagnoses in this study were derived from the NPR, which was established in 1977, and to which reporting is mandatory. Validity of the register is secured through quality control routines applied in the daily production and completion of annual reports [43,56]. Thirdly, The *CopDiff* database comes from a population which can be assumed to exhibit disease to a greater extent than the general population. The use of logistic regression analysis on the four-year incidence ensures that measures of excess risk (OR) can be interpreted independently of the frequency of the outcomes in the study. The OR is therefore a valid estimate for excess risk in the general population as well [57].

This study also has several limitations. Firstly, we were not able to identify the different causes of eosinophilia in the DIFFs. However, eosinophil-related organ damage may occur irrespective of the cause of eosinophilia which renders this information less important in the present study. Certainly, most patients in this study registered with eosinophilia have a reactive condition, because primary causes are so rare. Secondly, we performed risk analysis on *de novo* cases of potential end-organ disease as reported in the NPR. We cannot rule out, however, that some conditions have evolved since prior to blood sampling due to developmental latency of some disease entities. Likewise, we did not have information about drug treatment. Some types of drugs are known to cause eosinophilia [2] whereas others, especially glucocorticoids, are known to induce eosinophilic apoptosis [58]. Systemic glucocorticoids may be a relevant treatment in asthma, but not in heart conditions. And still the hormesis phenomenon is observed. A potential confounding effect of steroids should be a matter of interest in future studies. Thirdly, the NPR only holds information on individuals who have been in contact with secondary care and therefore patients exclusively managed in primary care are not included in present analyses. Lastly, we did not have access to clinical information about the patients, such as weight, smoking, alcohol consumption, exercise patterns, and family history of disease. These are associated with several types of cancer and certain benign conditions. The relation of these clinical variables to the eosinophil count is less clear and not examined in detail [59,60], or do not show any major direct association [61].

Eosinophils may cause organ dysfunction either by secretion from circulating cells of eosinophil constituents or by tissue invasion of the activated granulocyte [1,2,9,10]. The observed plateau of risks around 1 × 10^9^/l may indicate to the physician managing eosinophilia that the risk do not increase proportionate at higher counts (Fig. 2), which has been a dilemma previously. In addition, this study demonstrates that there is indeed an increased risk below median count of 0.16 × 10^9^/l for an increase in odds-ratio for the same medical diagnoses. Clinically, it means that a normal or even low count of eosinophils do not justify to rule out a risk for organ affection by eosinophils, but on the contrary may contribute to explain, why patients may have normal eosinophil counts in e.g. asthma or allergy and still have symptoms from lungs and skin. Most likely, the interpretation which may combine these results is a relation to eosinophil trafficking and dynamics. An alternative interpretation than extravasation of the low numbers of eosinophils in blood could be an inhibition of production, or destruction of eosinophils. However, there is no consequence of not having eosinophils in this context [62], why this interpretation cannot explain the observed association with end-organ symptoms (Table 1, Table 2 and Fig. 2).

## Authorship contributions and disclosure of conflicts of interest

OWB co-designed the study, collected, analysed and interpreted data and drafted the manuscript. VS analysed and interpreted data and performed the statistical analyses. HCH and BL analysed and interpreted data. CLA co-designed the study, collected, analysed and interpreted data. All authors revised the manuscript critically for important intellectual content, and approved the final version to be submitted. The study has received no financial support or other benefits from commercial sources and none of the authors have any financial interests, which could create potential conflicts of interest.

## Provenance and peer review

Not commissioned, editor reviewed.

## Ethical approval

No ethical approval was required.

## Sources of funding

The study has received no financial support or other benefits from commercial sources and none of the authors have any financial interests, which could create potential conflicts of interest. funding.

## Author contribution

OWB co-designed the study, collected, analysed and interpreted data and drafted the manuscript. VS analysed and interpreted data and performed the statistical analyses.

HCH and BL analysed and interpreted data.

CLA co-designed the study, collected, analysed and interpreted data.

All authors revised the manuscript critically for important intellectual content and approved the final version to be submitted. The study has received no financial support or other benefits from commercial sources and none of the authors have any financial interests, which could create potential conflicts of interest.

## Consent

Not applicable.

## Guarantor

Ole Weis Bjerrum (MD), and Christen Lykkegaard Andersen (MD).

## Registration of research studies

www.researchregistry.com UIN 4955.

## Conflicts of interest

No author has any conflict of interest.

## Declarations of interest

None.
