# Supplementary material for: Association of the blood eosinophil count with end-organ symptoms
Source: Ann Med Surg (Lond). 2019 Jul 9;45:11–8. doi: 10.1016/j.amsu.2019.06.015 (PMC6637252; doi:10.1016/j.amsu.2019.06.015)
Supplement: Multimedia component 1 [file mmc1.docx]

**Supplementary Table 1:** Outcomes as defined by the International Classification of Diseases version 10 (ICD-10)

| **Outcome** | **Disease entity/group** | **ICD-10 code(s)** |
| --- | --- | --- |
| **Cardiac disease** [11-17] | Pericardium | I30-I32 |
|  | Endocardium | I33 |
|  | Valve | I34-I39 |
|  | Myocardium | I40-I43 |
|  | Conduction | I44-I49 |
|  | Heart failure | I50 |
|  | Other heart disease | I51-I52 |
| **Skin disease** [36-40] | Dermatitis and eczema | L20-L30 |
|  | Urticaria and erythema | L50-L54 |
| **Neurological disease** [28-35] | Cerebrovascular disease | I63-I64 |
|  | Degenerative diseases of the nervous system | G30-G32 |
|  | Encephalopathy, unspecified | G934 |
|  | Polyneuropathies | G60-G64 |
|  | Mononeuritis multiplex | G587 |
|  | Paralytic syndromes | G81-G83 |
| **Gastrointestinal disease** [24-27] | Diseases of esophagus, stomach and duodenum | K20-K23, K25-K31 |
|  | Non-infective enteritis and colitis | K50-K52 |
|  | Diseases of liver | K72-K77 |
|  | Disorders of gallbladder, biliary tract and pancreas | K83, K85-K87 |
| **Respiratory disease** [18-23] | Chronic lower respiratory disease | J40-J47 |
|  | Respiratory disease principally affecting the interstitium | J80-J82, J84 |
|  | Pleural disease | J90-J94 |

s
